# Independent Large Scale Duplications in Multiple *M. tuberculosis* Lineages Overlapping the Same Genomic Region

**DOI:** 10.1371/journal.pone.0026038

**Published:** 2012-02-07

**Authors:** Brian Weiner, James Gomez, Thomas C. Victor, Robert M. Warren, Alexander Sloutsky, Bonnie B. Plikaytis, James E. Posey, Paul D. van Helden, Nicolass C. Gey van Pittius, Michael Koehrsen, Peter Sisk, Christian Stolte, Jared White, Sebastien Gagneux, Bruce Birren, Deborah Hung, Megan Murray, James Galagan

**Affiliations:** 1 The Broad Institute, Cambridge, Massachusetts, United States of America; 2 Division of Molecular Biology and Human Genetics, Department of Biomedical Science, Faculty of Health Sciences, National Research Foundation Centre of Excellence for Tuberculosis Research, Medical Research Council Centre for Molecular and Cellular Biology, Stellenbosch University, Tygerberg, South Africa; 3 Massachusetts Supranational TB Reference Laboratory, Center for Health Policy and Research, University of Massachusetts Medical School, Shrewsbury, Massachusetts, United States of America; 4 Mycobacteriology Laboratory Branch, Division of TB Elimination, National Center for HIV/AIDS, Viral Hepatitis, STD, and TB Prevention Centers for Disease Control and Prevention, Atlanta, Georgia, United States of America; 5 Medical Research Council, National Institute for Medical Research, London, United Kingdom; 6 Harvard School of Public Health, Boston, Massachusetts, United States of America; 7 Department of Biomedical Engineering and Microbiology, Boston University, Boston, Massachusetts, United States of America; Institut Pasteur, France

## Abstract

Mycobacterium tuberculosis, the causative agent of most human tuberculosis, infects one third of the world's population and kills an estimated 1.7 million people a year. With the world-wide emergence of drug resistance, and the finding of more functional genetic diversity than previously expected, there is a renewed interest in understanding the forces driving genome evolution of this important pathogen. Genetic diversity in *M. tuberculosis* is dominated by single nucleotide polymorphisms and small scale gene deletion, with little or no evidence for large scale genome rearrangements seen in other bacteria. Recently, a single report described a large scale genome duplication that was suggested to be specific to the Beijing lineage. We report here multiple independent large-scale duplications of the same genomic region of *M. tuberculosis* detected through whole-genome sequencing. The duplications occur in strains belonging to both *M. tuberculosis* lineage 2 and 4, and are thus not limited to Beijing strains. The duplications occur in both drug-resistant and drug susceptible strains. The duplicated regions also have substantially different boundaries in different strains, indicating different originating duplication events. We further identify a smaller segmental duplication of a different genomic region of a lab strain of H37Rv. The presence of multiple independent duplications of the same genomic region suggests either instability in this region, a selective advantage conferred by the duplication, or both. The identified duplications suggest that large-scale gene duplication may be more common in *M. tuberculosis* than previously considered.

## Introduction

Mycobacterium tuberculosis, the causative agent of most human tuberculosis, infects one third of the world's population and kills an estimate 1.7 million people a year. Until recently, all strains *of M. tuberculosis* were considered essentially genetically identical. Yet data from a number of groups suggests greater genetic diversity than originally expected [1], [2], [3], [4], [5], with possible phenotypic consequences ranging from differences in virulence to the emergence of drug resistance. Understanding the evolutionary forces that drive genome evolution within this group of pathogens would thus have significant implications for the development of diagnostics, the management of the emergence of drug resistance, and the development of new anti-tuberculosis drugs and vaccines [2], [6]. Yet a complete understanding of the dynamics of *M. tuberculosis* genome evolution is still lacking.

*M. tuberculosis* and other related members of the MTB complex (MTBC) which include the human adapted *M. africanum*, as well as several animal-adapted species including *M. bovis*, *M. pinnipedii*, *M. caprae*, and *M. microti*, are thought to have evolved from a single successful rapidly growing environmental saprophytic ancestor [7], following a recent evolutionary bottleneck around 35,000 years ago [8], [9]. One scenario proposes that the MTBC emerged as a slow growing offshoot from a smooth tubercle bacilli population, which includes *M. canettii* [9]. Within the MTBC, evolution characterized by substantial gene loss gave rise to the different host adapted species [10]. *M. tuberculosis* subsequently underwent population expansion and diversification into several major lineages linked to human population growth and migration [3].

The emergence of the MTBC appears linked to a decrease in the tempo of genome evolution. Strains of *M. tuberculosis* display a remarkably low sequence diversity as compared to other bacterial species [11]. The majority of differences between strains of *M. tuberculosis* are single nucleotide polymorphisms (SNPs) or small indels, with only a modest number of polymorphisms separating strains - ranging from less than 2300 for strains from difference lineages, to fewer than 200 for strains from the same lineage, with more than 80% comprising single nucleotides (genome.tbdb.org/annotation/genome/tbdb/ReseqStrainInfo.html [2]).

Larger scale polymorphisms have also been studied, but nearly all studies have reported predominantly deleted sequences - typically deletions of small genomic regions spanning part of a gene to several genes [5], [12], [13], [14]. Deletions are often associated with flanking repeats sequences that can serve as templates for homologous recombination. Moreover, in some cases, deletions have been associated with hot spots for the insertion of repeat elements [15], [16], [17]. The functional significance of such deletions is under debate. Due to the unique nature of many reported genomic deletions, it has been argued that most are neutrally evolving genetic traits that confer no selective advantage [4]. However, examining deletions based on gene content has revealed repeated independent deletions of the same genes, suggesting a possible selective advantage in some cases [1]. The predominance of gene loss reported is often assumed to be consistent with reductive evolution associated with a pathogenic and intracellular lifestyle.

Remarkable, in contrast to most other well-studied bacteria [18], [19], [20], [21], [22], *M. tuberculosis* strains display no evidence of lateral gene transfer (LTG) or substantial genome rearrangements. Although smooth tubercle bacilli exhibit clear indications of ongoing genetic exchange [9], and evidence indicates that LTG may have played a significant role the distant evolutionary history of Mycobacteria [23], [24], [25], [26], [27], within the MTB complex, evolution appears to have been strictly a clonal phenomenon [4], [28]. The lack of gene gain through lateral transfer can be explained as being due to lack of opportunity stemming from an obligate intracellular pathogenic lifestyle. Mechanistic barriers also exist and are possibly attributable to the unique cell wall properties of MTB.

The lack of lateral gene transfer and the lack of evidence for large scale genome rearrangement events have led to the conclusion that large scale recombination has played a minimal role, if any, in the evolution of *M. tuberculosis*. However, recent evidence has reopened this question. A publication by [29] reported the duplication of a 350 Kb region, spanning Rv3128c to Rv3427c, in two strains belonging to the W/Beijing family of *M. tuberculosis* lineage 2. When PCR was used to assay for duplications arising from the same junctions in other strains, the identical duplication was identified in multiple strains in 4 of the 5 Beijing groups, but not in any non-Beijing strains. The authors suggest that the identified duplication is restricted to the most recently evolved Beijing strains [29], and may lead to a dosR overexpression phenotype.

We report multiple independent occurrences of a large-scale duplication of the same genomic region of *M. tuberculosis* detected through whole-genome sequencing. The duplications overlap or are congruent with the duplications recently reported [29], and are found in both drug susceptible and resistant strains from *M. tuberculosis* lineages 2 and 4, in addition to the Beijing strains. The duplicated regions also have substantially different boundaries in different strains, indicating different originating duplication events. We further identified a smaller segmental duplication of a different genomic region of a lab strain of H37Rv. The presence of multiple independent duplications of the same genomic region suggests either an instability in this region, a selective advantage conferred by the duplication, or both. The identified duplications suggest that large-scale gene duplication may be more common in *M. tuberculosis* than previously considered.

## Results

### Sequenced strains and strain phylogeny

We report 4 newly sequenced *M. tuberculosis* strains - M73, M41, M141, and CDC606 - from different lineages with a range of drug resistance profiles (Table 1). These strains were part of a larger project encompassing 46 strains with a range of drug resistances, from disparate geographic locations, and belonging to lineages 3 and 4. In addition, we re-sequenced a lab strain of a lab strain of H37Rv [30]. We also report the re-analysis of T67, available from the TB Diversity Sequencing Project ([6], [31], data available at http://genome.tbdb.org/annotation/genome/tbdb/ReseqStrainInfo.html).

**Table 1:** Strains Displaying Duplications.

*Strain*	*Lineage*	*Resistance Profile*	*Source*
M41	MTB 4	Inh,Rif,Str,Kan,Emb,Ethi,Oflox	Stellenbosch
M141	MTB 4	Inh,Rif,Str,Kan,Emb,Ethi,Oflox	Stellenbosch
CDC606	MTB 2 - Beijing Group 3	Inh,Rif,Str,Kan,Pza,Tha,Emb	CDC
T67	MTB 2 - Beijing Group 4	DS	[2]
M73	MTB 4	Inh	Peru
H37Rv Lab	MTB 4	DS	Hung Lab

Inh: Isoniazid,; Rif: Rifampin; Str: Streptomycin; Kan: Kanamycin; Eth: Ethionamide; Oflox: Ofloxaxcin; Tha: Tacrine; Emb: Ethambutol; Pza: Pyrazinimide; DS: Drug susceptible.

**Figure 1:**
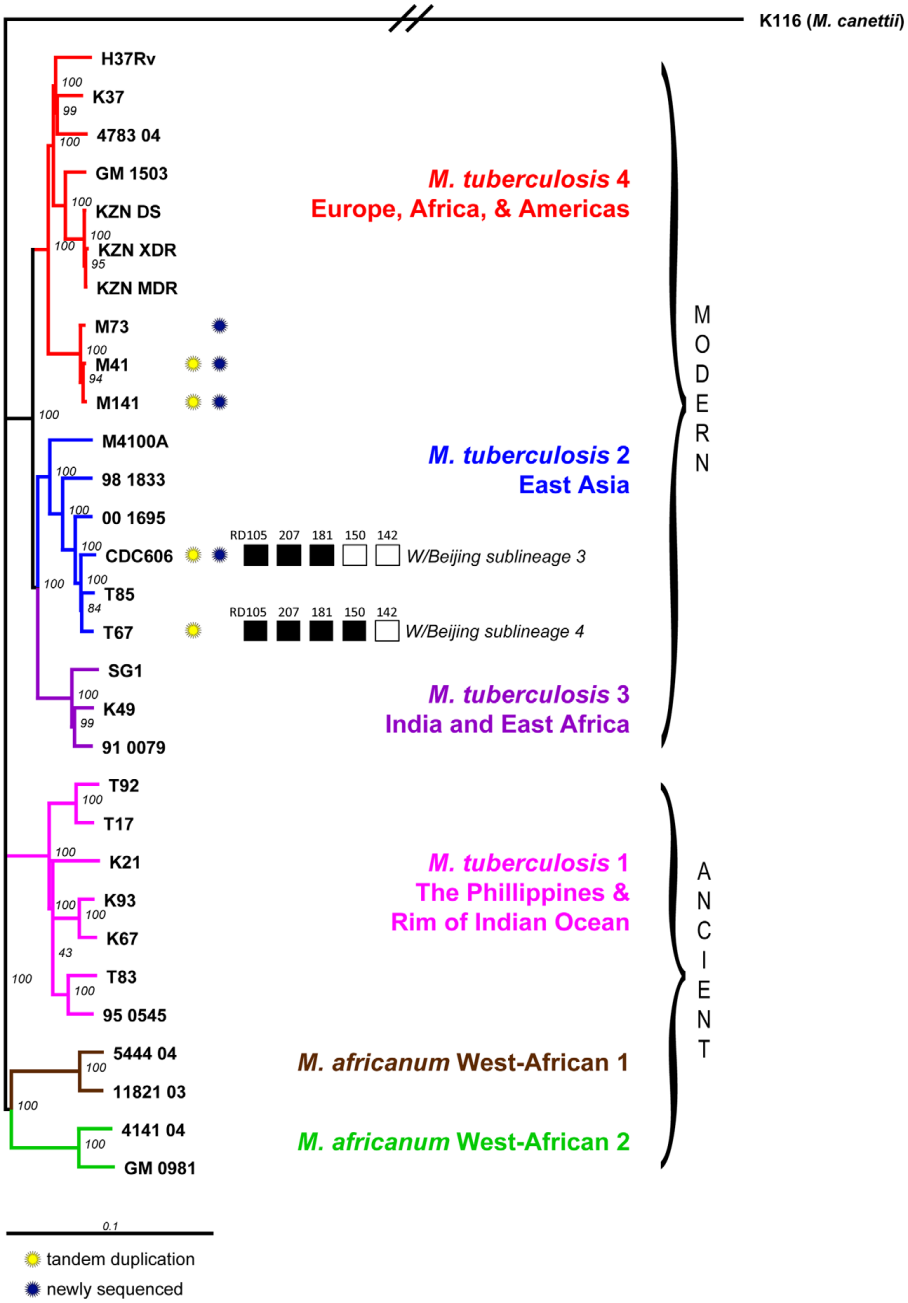
Phylogeny of sequenced strains in the context of the global TB phylogeny. Strains marked with a blue circle were sequenced as part of this project. The remaining strains were sequenced previously as part of the TB Diversity Sequencing Project. Strains marked with a yellow circle contain the large segmental duplication near 3.7 Mb. For CDC606 and T67, the results of a digital spoligotype indicate the Beijing group to which these strains belong.

To place the strains in the context of the global MTB phylogeny, we performed a phylogenetic analysis using polymorphisms relative to the reference genome of H37Rv (see Methods). The resulting phylogeny is shown in Figure 1. according to which M41, M141, and M73 belong to MTB lineage 4/European & American (along with H37Rv), while CDC606 and T67 belong to MTB lineage 2/East Asian which includes the Beijing/W group strains.

To further classify the precise genetic lineage of CDC606 and T67 we examined regions for deletions characteristic of different Beijing family groups (see Methods). The presence of deleted region 105 defines a strain as Beijing/W whereas the presence/absence of the remaining deleted regions defines the specific subgroup. A region was considered deleted if substantial coverage (more than 80% of region) was not observed and had no coverage at the beginning and end of the junction (Figures S1, S2, S3, S4, S5 S6). The presence or absence of particular regions is shown as a digital spoligotype in Figure 1.

### WGS reveals multiple independent duplications of the same large genomic region

**Figure 2:**
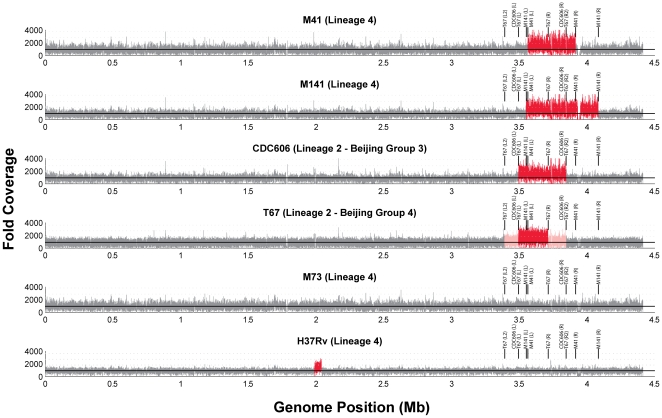
Coverage of sequenced strains relative to H37Rv. Each plot shows the fold coverage for the alignment of each strain to H37Rv. The mean coverage for each strain is denoted by the solid horizontal line, while multiples of the mean are denoted by the horizontal grey lines. For most of the genome the coverage varies around the mean coverage (grey). Duplicated genomic segments are apparent as regions varying around twice the mean coverage (red). T67 also contains regions with coverage between 1× and 2× the mean (pink). The boundaries of each duplicated region are shown as vertical lines in each plot.

Sequencing was performed on the Illumina GAIIx using both 36 bp unpaired reads and 70 bp paired reads (see Methods). Sequence coverage at each base - defined as the number of times each base was sequenced - was calculated by aligning reads to the genome sequence of H37Rv and calculating the number of reads aligned over each position. As expected, coverage for all strains varies around the mean coverage for the majority of the genome (Figure 2 - grey). Strains M41, M141, CDC606, and T67, however, display a large contiguous segment with coverage twice the mean coverage for the genome (Figure 2 - red), reflecting an apparent large scale genomic duplication of this region in all 4 strains.

Remarkably, the duplication in all 4 strains occurs in the same genomic region near 3.7 Mb. Moreover, this region overlaps the duplications in the Beijing strains reported by [29]. Although CDC606 and T67 are both members of *M. tuberculosis* lineage 2, which contains the strains reported in [29], strains M41 and M141 are members of *M. tuberculosis* lineage 4. Thus, a large scale duplication overlapping the same genomic region has occurred independently in at least two lineages and is not limited to Beijing strains.

The lack of duplications in sequenced phylogenetic near neighbors supports the conclusion that these duplications are independent events. Strain T85, a near neighbor of T67, was previously sequenced for the TB Diversity Sequencing Project and does not display a duplication (data not shown). Strain M73 is a near neighbor of M41 and M141, while H37Rv is a more distantly related strain in lineage 4. We sequenced both M73 and a lab strain of H37Rv for this project, and neither strain displays a duplication of the region around 3.7 Mb (Figure 2). Of note, the phylogeny of strains T85, CDC606, and T67 places T85 as a sister strain of T67, and CDC606 as an out-group of these three strains. This suggests either that T85 contained and lost the duplication, or that the two duplications in CDC606 and T67 are independent events. The close correspondence between the duplication boundaries, and the instability of the duplications previously reported [29] would possibly argue for the latter.

### Boundaries suggest a common origin for duplications in CDC606 and T67

The duplications in CDC606 and T67 show a close correspondence to one another and to the segmental duplication in the Beijing strain reported in [29]. Both CDC606 and T67 as well as the strains with reported duplications in [29] are members of the W/Beijing family of *M. tuberculosis* lineage 2 (Figure 1). Based on digital spoligotyping, CDC606 belongs to group 3 of the W/Beijing family, and T67 belongs to group 4. In addition, both the duplications in CDC606 and T67 share a common upstream boundary between Rv3127 and Rv3128c, matching the duplications reported in [29]. The duplication in CDC606 also matches the downstream boundary of the duplications in [29], occurring between Rv3427c and Rv3428c.

Curiously, the regions adjacent to both sides of the duplication in strain T67 display coverage elevated with respect to mean coverage for the genome as a whole, but less than the 2-fold of increase evident in the core duplicated region (pink regions in Figure 2). We refer to these regions as the partially duplicated regions of T67. The coverage between 1× and 2× the genomic mean suggests that the sequenced population of bacteria for this strain was not clonal, and a proportion contained a larger duplication in this region (the L2 boundaries in Figure 2).

Interestingly, the downstream boundary of the partially duplicated region in T67 - between gene Rv3427c and Rv3428c, corresponds to the downstream boundary of both CDC606 and the duplicated strains of [29]. In contrast, the upstream boundary of the partially duplicated region in T67 lays 110 genes upstream of the boundary in CD606 at roughly Rv3048c.

The correspondence between duplication boundaries in CDC606, T67, and the strains reported in [29] supports the idea of a single duplication occurring within the W/Beijing family of lineage 2, with a downstream boundary at Rv3427c, that was subsequently inherited or lost in other Beijing strains. The loss of duplicated regions is consistent with the instability of duplications in vitro reported by [29]. The partially duplicated downstream region in T67 ending at Rv3427c, the boundary noted in other Beijing strains, is consistent with this model. The partially duplicated upstream region and T67 is more curious. This may reflect a larger duplication occurring either specifically in MTB lineage 2 or at a deeper ancestor with subsequent loss in other strains, or possibly a second duplication event.

### Boundaries suggest multiple origins for duplications in M41 and M141

In contrast to CDC606 and T67, the duplication boundaries for strains M41 and M141 differ considerably from each other. The duplication in strain M41 spans the region from Rv3193c to Rv3495c while the duplication in M141 spans Rv3188 to Rv3644 (Table 2). At 532 Kb, the duplicated region in M141 is the largest segmental duplication in *M. tuberculosis* reported to date.

**Table 2:** Summary of Large Scale Duplication Boundaries.

*Strain*	*Upstream Boundary* *	*Downstream Boundary* *	*Size (Kb)*
M41	Rv3193c (3,561,951)	Rv3495c (3,914,339)	352.4
M141	Rv3188 (3,549,789)	Rv3644 (4,082,013)	532.2
CDC606	Rv3128c (3,493,080)	Rv3427c (3,844,681)	351.6
T67§	Rv3128c [Rv3048c]	Rv3326 [Rv3427c]	∼281 [∼350]
Domenech [29]	Rv3128c	Rv3427c	∼350
Resequenced H37Rv	Rv1756c	Rv1800	53.6

*Numbers in parenthesis are nucleotide positions for the duplication boundaries derived from PCR amplification.

**Figure 3:**
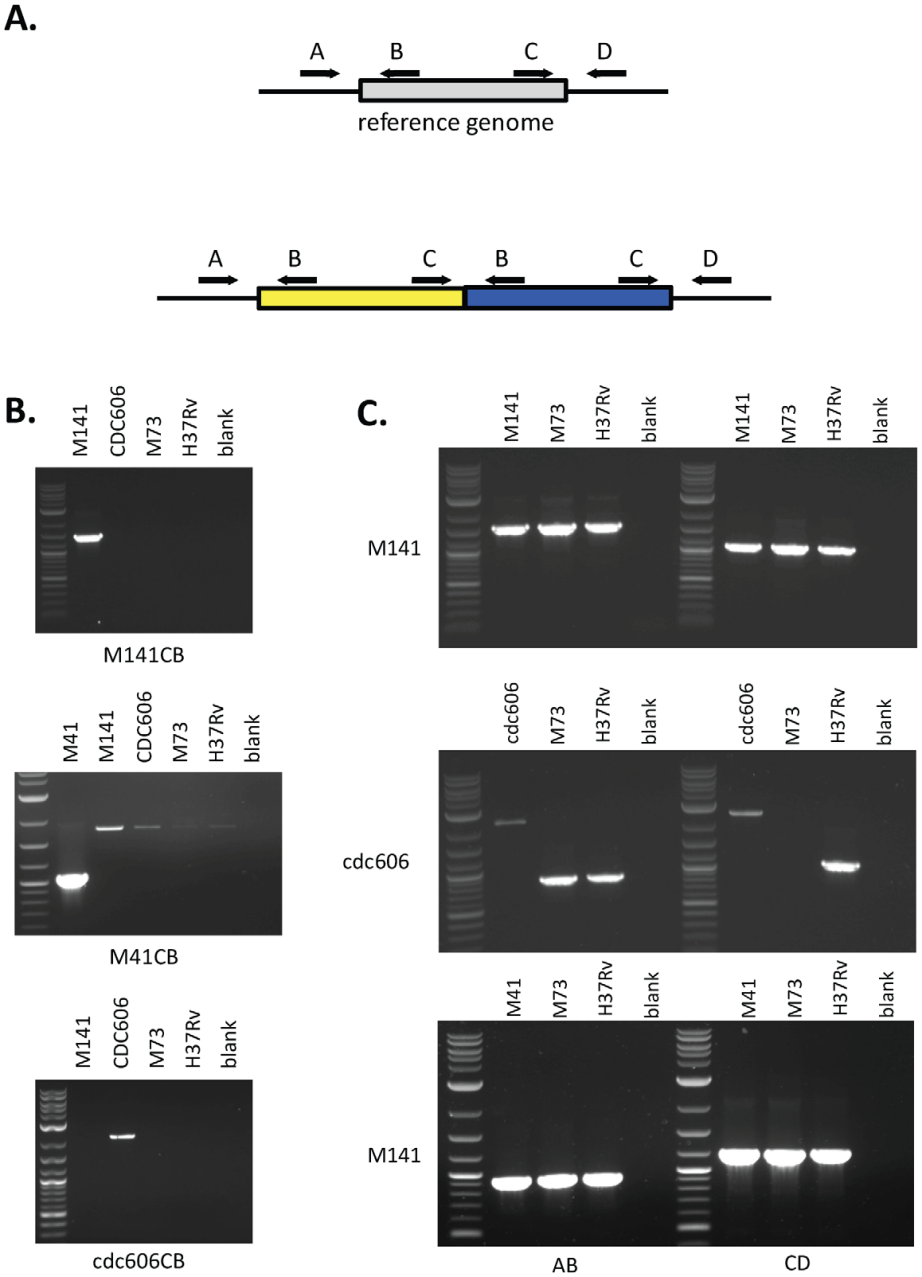
PCR verification of tandem duplications. (A) Schematic representation of primer design strategy. The duplicated region is shown as a grey box, present as a single copy in the reference genome (top) and as a tandem duplication below. Primers A and B amplify the left flank and C and D amplify the right flank. Only when C and B and brought into close proximity by a tandem duplication can a product be generated using primers C and B. (B) 0.9% agarose gel loaded with 5 ul of PCR reactions using primers C and B specific for isolate M141 (top), M41 (middle) and cdc606 (bottom). Lane 1 contains the 2 log ladder (New England Biolabs, N3200). (C) 0.9% agarose gel loaded with 5 ul of PCR reactions using primers A and B (lanes 2–5) or C and D (lanes 7–10) specific for each isolate as indicated on the left. Lanes 1 and 6 contain the 2 log ladder (New England Biolabs, N3200).

§ Genes in square brackets correspond to the partial duplication boundaries for T67 (L2 and R2 in Figure 3) - see text for details.

The markedly different boundaries between M41, M141, and the other strains reported here and in [29] suggest the possibility that two independent duplication events occurred in M41 and M141 to give rise to their segmental duplication. However, we cannot rule out the possibility that only a single duplication event occurred in an ancestor of M41 and M141, with a portion of the duplication subsequently lost in M141 to produce the shorter upstream boundary. In either scenario, the data suggest independent duplications in lineages 2 and 4 using different duplication boundaries.

### PCR confirmed tandem duplications and reveals precise junctions

To gain insight into the location of the two copies of each of the duplicated regions, we examined the alignment of read pairs near the junctions of the duplications in M41, M141, and CDC606 (paired reads were not available for T67). For strains M41 and M141, a clear picture of a tandem duplication emerged. For each duplication, reads near the ends of each duplication had read pairs that aligned to one of two locations: the neighboring single copy region or the other end of the duplication. Although our data did not reveal as clean a pattern for CDC606, the confirmation of a tandem duplication by [29], and the correspondence of that duplication to the one in CDC606 led us to hypothesize a similar arrangement in this strain.

**Figure 4:**
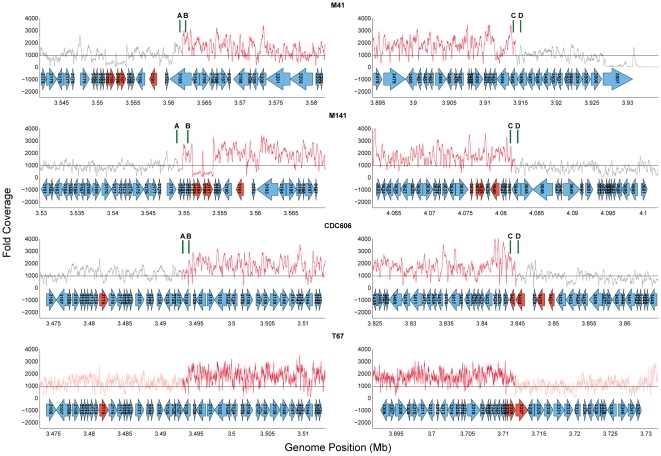
Coverage plots around duplication boundaries. Each plot shows the fold coverage for the alignment of the corresponding strain to H37Rv around the upstream (left column) or downstream (right column) duplication boundaries. Color coding for coverage plots is as in Figure 2. Genes in red are predicted repeats or transposable elements in H37Rv. Vertical green bars indicate the positions of PCR primers used (see Table 3).

To verify that tandem duplications had occurred in strains M141, M41, and CDC606 and clarify the precise structure of their junctions, we used polymerase chain reaction (PCR) to amplify the relevant regions of these genomes. For each strain, four primers were designed: two pairs (A&B, C&D, Figure 3A), each of which flanks the ends of the duplicated region. In each pair, one primer originates from within the duplicated region (B and C) while the other lies just outside of the predicted duplication (A and D); both pairs were predicted to yield a product in all strains, regardless of the presence of a duplication. However, in the presence of a tandem duplication, primers B and C would together generate an amplicon that spans the junction of the tandem duplication. Using this strategy, primers were designed for M141, M41 and CDC606, and these were used to amplify the regions described above (Figure 4, Table 3).

**Table 3:** Primer locations for PCR Confirmation.

*Name*	*Sequence*	*Location (H37Rv)*
M141A3	CTAGTCGACGCGCTATTCAA	3548991-3549010
M141B3	GCAGCTCCTTCATGTTGTGA	3550545-3550526
M141C2	GCCATTAAAGAGCAACAGGC	4081312-4081331
M141D2	CAGATCGTGGTGATTGAGGA	4082355-4082336
M41A5	CGCTTCCGGTGTAGGTGTAT	3561576-3561595
M41B6	GCTTATTGGCTGGATCGGTA	3562497-3562478
M41C3	CCAGGTTGTCCTTGTCACG	3913856-3913874
M41D3	ACCAGAGCGAGAACATCGAG	3915033-3915014
CDC606 A3	ATAGAAATGCCCGTGAAACG	3493141-3493160
CDC606 B3	CGAAGGCCAAAGACCAGATA	3494003-3493984
CDC606 C3	GGAACAGGCCGTACCAGTTA	3844001-3844020
CDC606 D3	ACCGACTTCTCCCACTACCC	3845085-3845066

A, B, C, and D are as indicated in Figure 3 and also described in text.

**Figure 5:**
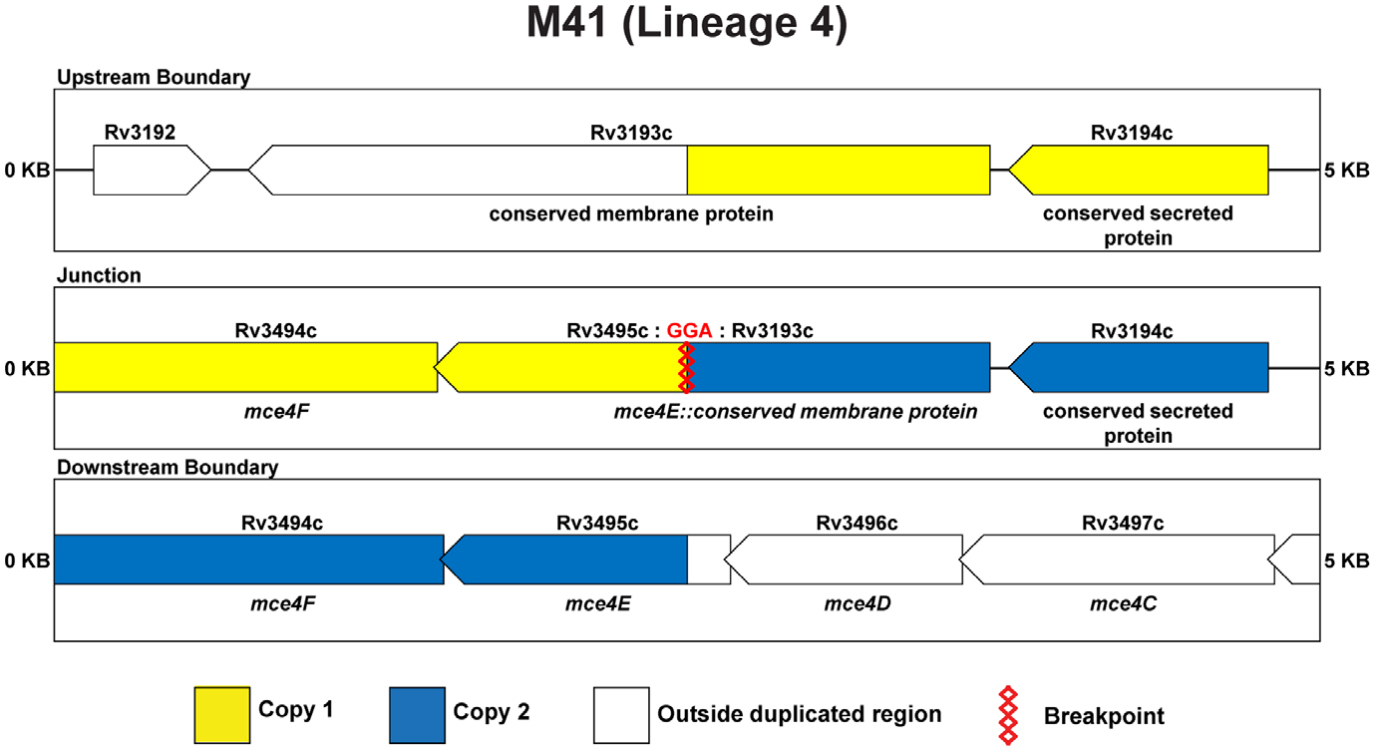
Schematic of duplication boundaries and junctions for M41. Genes within the tandem duplications are color coded. Genes in yellow are present in the upstream duplication copy, while genes in blue are present in the downstream copy.

For strain M41, as can be seen in Figure 3B, the BC primer pair generated a 1028 bp amplicon unique to this isolate. Sanger sequencing demonstrated that the repeated region corresponds to bases 3,561,951 through 3,914,339, with the junction defined by a 3 bp sequence (GGA) present on both ends of the duplicated region that is present in just one copy at the fusion (Figure 5).

**Figure 6:**
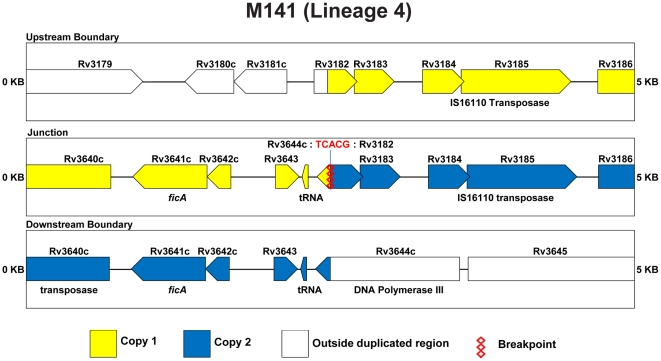
Schematic of duplication boundaries and junctions for M141. Color coding is identical to Figure 5.

Similarly, for strain M141 a 1.45 kilobase (kb) amplicon was generated from genomic DNA using the CB primer pair that spanned the junction of the duplication, and sequencing of this amplicon confirmed the proposed structure – a direct repeat of the region corresponding to bases 3,549,789 through 4,082,013 of the H37Rv chromosome, with the junction defined by a 5 bp sequence (TCACG) present on both ends of the duplicated region that is present in just one copy at the fusion (Figure 6). This region was not amplified from CDC606 the related isolate M73, or the H37Rv reference strain; the AB and CD primer pairs yielded products of the expected size from all strains tested (Figure 3C).

**Figure 7:**
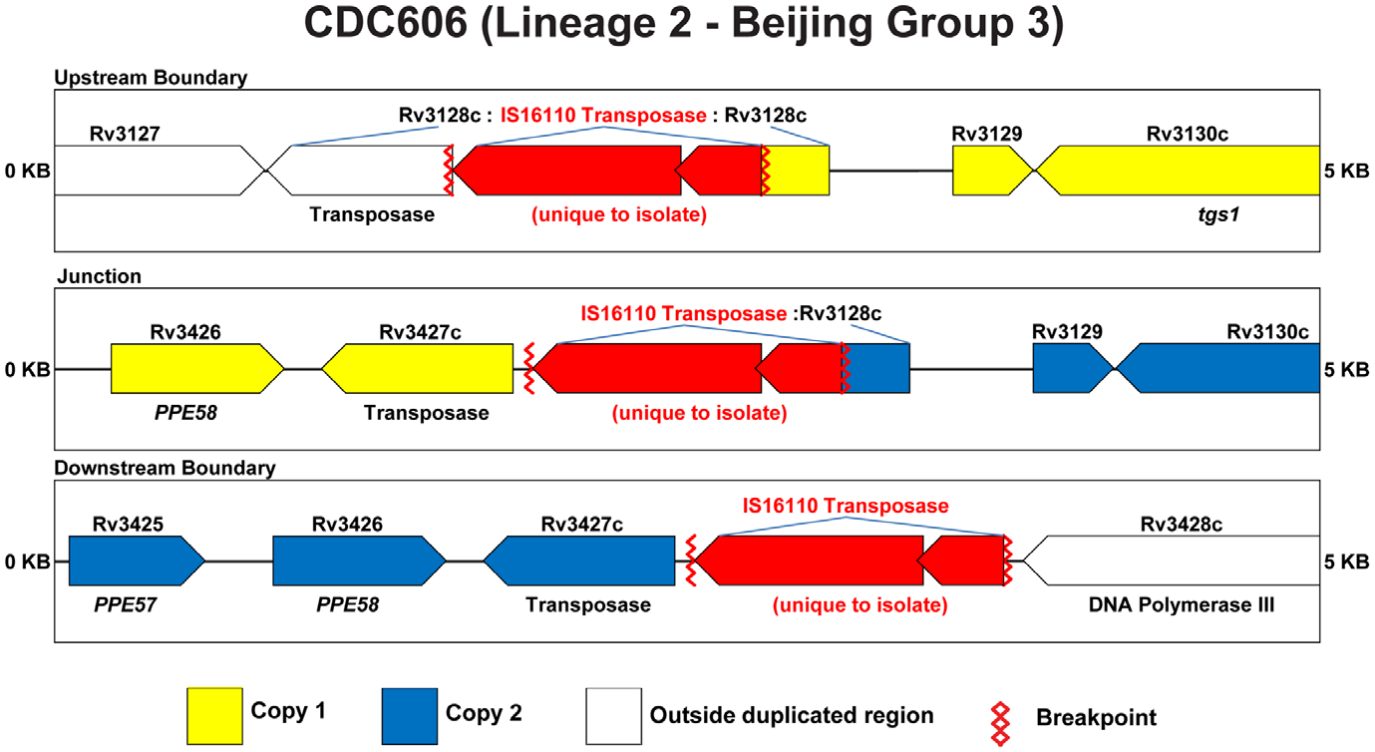
Schematic of duplication boundaries and junctions for CDC606. Color coding is identical to Figure 5 with the exception that genes present in strain CDC606 but missing in the reference genome sequence for H37Rv are colored in red.

Finally, for strain CDC606, the CB primer pair yielded a 2.14 kb amplicon spanning the junction of the duplicated regions that was not detected in any of the other strains tested (Figure 3B). Sequencing of this amplicon showed that the junction of the tandem duplication (bases 3,493,908 through 3,844,681 of the H37Rv chromosome) includes a complete copy of the IS*6110* insertion element. IS*6110* is also present at either end of the duplicated region, though absent in the reference strain H37Rv (Figure 7). The presence of this strain specific insertion elements at the boundaries and junctions of the duplication in CDC606 is identical to the configuration reported in [29], further supporting the conclusion that this represents a single event in the Beijing lineage.

### Duplicated region intersection enriched for purine and pyrimidine metabolism, DNA metabolism, and repair

The genes involved in the segmental duplications may provide a clue to the selective advantage, if any, that the duplications confer. The different duplication boundaries result in different genes being significantly enriched in the different duplications. For example, CDC606 displays a unique enrichment for genes annotated as energy production and conversion, while the M41 duplication is enriched for genes implicated in lipid metabolism. Yet a common set emerges when we consider the intersection of the duplicated regions (Data S1). The intersection of all duplications appear enriched for genes involved in purine and pyrimidine metabolism, as well as genes with roles in DNA repair (Table 4). Specifically, four helicases are located in the duplication intersection, including three UvrD helicases. UvrD helicases unwind and displace DNA near the site of mismatches during repair. Although the duplicated regions do contain several transposase genes, the regions are not significantly enriched for transposases relative to the rest of the genome - calculated either using the union or intersection of the duplicated regions.

**Table 4:** Genes Involved in Nucleotide Metabolism and DNA repair enriched in the intersection of all duplicated regions.

Locus	*Gene Name*
Rv3198c	ATP-dependent DNA helicase II uvrD2
Rv3199c	NADH pyrophosphatase nudC
Rv3201c	ATP-dependent DNA helicase
Rv3202c	ATP-dependent DNA helicase
Rv3204	DNA-methyltransferase
Rv3210c	conserved hypothetical protein
Rv3211	ATP-dependent RNA helicase rhlE
Rv3247c	thymidylate kinase tmk
Rv3263	DNA methylase
Rv3275c	phosphoribosylaminoimidazole carboxylase catalytic subunit purE
Rv3276c	phosphoribosylaminoimidazole carboxylase ATPase subunit purK
Rv3297	endonuclease VIII nei
Rv3307	purine nucleoside phosphorylase deoD
Rv3309c	uracil phosphoribosyltransferase upp
Rv3313c	adenosine deaminase add
Rv3314c	thymidine phosphorylase deoA
Rv3315c	cytidine deaminase cdd
Rv3370c	DNA polymerase III alpha chain dnaE2
Rv3393	nucleoside hydrolase iunH
Rv3394c	conserved hypothetical protein
Rv3396c	GMP synthase guaA
Rv3410c	inosine-5-monophosphate dehydrogenase guaB3

### No evidence of increased mutation rate within duplicated regions

Genome duplication provides a mechanism for generating new gene function by creating copies of genes such that one or both copies are free to diverge and acquire novel functions [18], [32], [33], [34]. To determine if genes within the tandem duplications appear to be evolving more rapidly, or acquiring different mutations within copies of the duplication, we analyzed single nucleotide polymorphisms (SNPs) in M41 and M141 within the duplicated regions relative to the unduplicated genomic regions.

SNPs were detected by aligning reads to the reference genome sequence for H37Rv (see Methods). To differentiate polymorphisms associated with lineage differences, we identified SNPs present in M141 and M41 but not present in M73, a closely related strain that lacked the duplication. Our analysis revealed no evidence for an increase in mutation rate within the duplicated regions (Data S2).

Furthermore, we see no clear evidence for mutations occurring in only one copy of the duplication. To attempt to detect such cases, we examined positions in which roughly 50% of reads indicate a base identical to the cousin strain, and roughly 50% differed. In M141, several such instances were detected, but all fell within transposases or PE/PPE genes. In M41, only two instances were detected - one in a transposase and one in a PPE gene (Data S3). Given the repetitive nature of these genes, these instances likely represent alignment artifact rather than true polymorphisms.

### Sequencing of H37Rv lab strain predicts an additional segmental duplication

As indicated above, to check for the presence of the large segmental duplication near 3.7 Mb, we re-sequenced a lab strain of H37Rv (see Material and Methods). As expected, we did not see a large segmental duplication near 3.7 Mb. But we were surprised to identify a small segmental duplication in the sequenced H37Rv strain spanning 53 kb from Rv1756c to Rv1800. Single nucleotide polymorphisms have been previously reported in lab passaged strains of H37Rv relative to the genome sequence strain. Our data indicate that in addition to SNPs, segmental duplications can also occur, and may be more common than previously expected.

## Discussion

Recombination and large scale genome rearrangements are generally thought to play a minimal role if any in the evolution of *M. tuberculosis* strains. We report here multiple independent occurrences of a large-scale duplication of the same genomic region of *M. tuberculosis* detected through whole-genome sequencing. The duplications overlap or are congruent with the duplications recently reported [29].

The newly sequenced strains reported here were part of a larger sequencing project encompassing 46 strains with a range of drug resistance profiles, from disparate geographic locations, and belonging to lineages 3 and 4. In additions, the strains re-analyzed from the TB Diversity Sequencing Project [6], [31] encompassed over 22 genomes from all 4 lineages. Within this set of genomes, only the 5 strains reported here harbored large scale duplications, and in each case the duplication occurred in the same genomic location.

The occurrence of multiple independent duplications in different lineages and overlapping the same genomic region is noteworthy. These data suggest that this particular genomic region is either more prone to duplication than the rest of the genome, confers a selective advantage when duplicated, or both.

### Duplications overlap duplicated region in BCG

Remarkably, the duplication reported here and in [29] matches a region that has also been duplicated in *M. bovis* BCG Pasteur [35]. Brosch and colleagues describe two tandem duplications, DU1 and DU2, in BCG relative to *M. tuberculosis* H37Rv through the analysis of BAC clones. DU1 encompasses the region from Rv3910 to Rv0013 (and thus includes the origin of replication). This region was not duplicated in any of the strains that we analyzed. In contrast, the DU2 region did overlap the large-scale duplication in our report. Based on the analysis of Brosch and colleagues, DU2 arose from a duplication of the region from Rv3214c to Rv3302c. Subsequently an internal deletion removed the segment from Rv3231c to Rv3291c in one copy of the original duplication leaving a residual 36 Kb duplicated regions. This residual region was then duplicated again in a subpopulation of BCG Pasteur.

As the genomes of *M. bovis* BCG and *M. tuberculosis* are highly similar in the region around DU2 and the large scale duplication that we and Domenech et al. report, the report by Brosch et al. further supports the conclusion that this region is particularly susceptible to undergoing or maintaining tandem duplications. Moreover, the finding from Brosch et al. that the DU2 region incurred both an internal deletion and subsequent additional deletion is consistent with the instability of duplications reported by Domenech et al., and the apparent partially duplicated regions we observed in strain T67.

The duplicated region may be prone to instability due to specific physical features. For example, repeat sequences play a role in enabling rearrangement through homologous recombination [36]. Although the duplicated regions do contain a number of annotated transposases in H37Rv, this region is not statistically enriched for such repeat elements relative to the rest of the genome. Nor does this region correspond to previously identified hot spots for the insertion of mobile elements in *M. tuberculosis* such as the DR or ipl regions [15], .

Nonetheless, the presence of IS*6110* elements in the duplicated region of CDC606 and the strains reported by [29], do provide a mechanism for duplication in these instances (see below). In this case, homologous recombination between IS*6110* elements flanking the duplicated region appears to be the likely mechanism for the duplication. In the case of strains M41 and M141, PCR and sequencing did not reveal the presence of transposons at the duplication boundaries. Instead, small duplicated sequences were present at both the boundaries and tandem duplication boundaries. It is possible that these small duplicated sequences were sufficient to support homologous recombination [38]. It is also possible that the duplication was mediated by repeat elements that were lost with subsequent decay of the duplicated regions. The latter possibility is consistent with the instability of the duplicated regions reported by [29].

It is also formally possible that the rest of the genome is resistant to duplication. The duplication in the lab strain of H37Rv, however, would argue against this. In addition, the unrelated duplication in BCG reported by [35] also argues against this. Moreover, unrelated genome duplications and rearrangements are frequent in related Mycobacteria [23], [39], [40], which, though they differ from *M. tuberculosis* more substantially, also display significant gene conservation. Finally, the ability to episomally clone genes into *M. tuberculosis* also suggests that many genes outside the duplicated region can tolerate gene copy number increases.

### The duplications may confer a selective advantage

The repeated occurrences of duplications of the same genomic region may also suggest possible functional consequences that carry a fitness advantage. For example, it is possible that changes in gene copy number may lead to increases in gene expression for genes within the duplicated regions. Alterations in the copy number of key regulators could also lead to changes in gene expression for genes outside the duplicated regions. We have not verified changes in gene expression associated with the genome duplication events.

With respect to the genes involved in the duplications, the size of the duplications results in a large number of candidate genes for which gene copy changes might result in functional changes. A large number of well studied genes with implications for virulence, pathogenesis, and drug resistance are present in the duplications, and it is thus possible to generate a large number of hypotheses. With respect to genes statistically enriched in the duplicated regions, it is perhaps interesting to note that the genes common to all the duplications are enriched for DNA metabolism and DNA repair. It is highly speculative, but possible, that this particular region is more liable to duplication as it contains genes that enable the organism to more efficiently scavenge nucleotides for subsequent DNA replication of the additional nucleotides, though studies in other organisms suggests that duplicated DNA carries little appreciable metabolic cost [41]. It should be noted that statistical enrichment is certainly not required for a gene copy number change to have functional significance. A single gene change may be sufficient. Although the large size of the independent duplications suggests that if there is a functional significance, it may involve a set of genes dispersed throughout this region.

Finally, gene duplications are generally considered to play a role in the evolution of new gene functions [18]. In particular, when a gene is duplicated, one or both copies of the gene may undergo accelerated evolution [42]. Furthermore, the presence of multiple copies of the same duplicated sequence can result in further homologous recombination leading to further genome evolution. Nonetheless, we are unable to detect any acceleration of DNA evolution in the duplicated regions. Although SNPs are present throughout the duplicated regions relative to H37Rv, when filtered for changes that are specific to the duplicated strains (by comparison with a closely related unduplicated strain), few polymorphisms are evident. Apart from polymorphism in transposons, nearly all SNPs are found in PE and PPE genes. The repetitive nature of all these genes, which can lead to substantial artifacts in aligning sequence reads, makes such detected polymorphisms suspect.

A number of possible explanations may exist for the lack of more apparent nucleotide changes. The most likely explanation is that there has been insufficient time since the duplication events in order for sufficient mutations to have occurred to allow us to detect differences. This is particularly likely given the overall slow mutation rate of *M. tuberculosis*. It is also possible that the particular genes within the duplicated region remain under the same purifying selection even in a duplicated state.

### No clear association with drug resistance

The strains containing the large scale duplications include both drug resistant and susceptible strains (Table 1). With the exception of T67, the strains reported in this study were all selected for their drug resistance phenotype. Three of the strains reported to contain the large scale duplication are resistant to multiple first and second line drugs. In contrast, T67 and the strains reported in [29] are not reported to be resistant and we verified sensitivity to first and second line drugs (see Methods). Thus the duplicated regions to not appear to be associated with drug resistance. In addition, the finding that this region has also duplicated in BCG [35] further suggests that drug resistance is not the primary factor.

The role of large scale rearrangements in the genome evolution of the *M. tuberculosis* is poorly understood. Although it has long been thought that large recombination events play little or no role in sculpting strain differences, the recent report by [29] makes clear that such events are possible. With the strains studied in this report, we establish that the large scale duplications are not limited to Beijing strains and that multiple independent duplications have occurred, likely with different boundaries, in at least two *M. tuberculosis* lineages. Whether the duplications around position 3.7 Mb reflect genome instability of this region or a selective advantage associated with copy number increases for genes in this region, the multiple independent occurrences in two different lineages indicates a disposition for this region to rearrange. Yet large scale duplications are clearly not limited to this region as we further identified a smaller segmental duplication of a different genomic region in a lab strain of H37Rv. Together these data suggest that large scale duplications in *M. tuberculosis* may be more common than assumed. Whether such events play a role in differences between strains with respect to virulence, pathogenesis, or drug resistance remains to be determined. However, the increasing availability of whole genome sequence continues to shed light on the role of genome rearrangements in general, including transpositions and inversions, in the evolution of this unique pathogen.

## Materials and Methods

### Phylogenetic analysis

A superset of SNPs relative to the canonical laboratory strain of H37Rv [43] were created across all clinical isolates from the Maq SNP reports. Repetitive elements including transposases, PE/PPE/PGRS genes, and phiRV1 members (273 genes, Data S4) resulting in non-unique 36mers were removed from the SNP reports due to the inability of Illumina sequencing to discriminate between multiple identical sites in the genome [2]. Furthermore, an additional 39 genes associated with drug resistance [44] were also removed to determine if classic drug resistance mutations were significantly altering the relatedness of the phylogeny (Data S4). The superset of 21,647 SNPs were concatenated and the phylogenetic tree determined with PhyML v3.0 [45] to first build a BIONJ tree using *M. canettii* as the root. The resulting phylogeny was then used as the guide tree for subsequent bootstrap analysis to determine the confidence of the branchings.

### Genome sequencing

Strains CDC606, M141 and M41 were sequenced by the Illumina GAIIx using a combination of paired and unpaired reads. Each strain received one lane of 36 bp unpaired reads and one lane of 76 bp paired reads. Strain T67 was sequenced on a lane of Illumina at 50 bp read length, and Sanger sequencing was also carried out; pOTs and pJANs to a coverage of 8×, as previously described [2]. All strains were cultured in standard 7H9 media lacking glycerol.

### Read alignment and coverage calculation

Sequence reads were all aligned to the reference genome sequence for H37Rv using Maq [46]. Each read of a read pair was aligned independently. Reads that aligned with more than 4 mismatches or that aligned to multiple locations were discarded. Sequence coverage at each base was calculated as the number of reads aligned over each position. Coverage was plotted and analyzed using custom Matlab scripts.

### PCR verification of tandem duplications

One to ten nanograms of genomic DNA were used as a template for PCR. PCR reactions were carried out using Expand High Fidelity PCR System (Roche, 11 732 641 001) using buffer 2, 0.5 um of each primer, 200 uM dNTPs, and 5% DMSO. Reaction conditions included an initial 5-minute denaturation step at 95°C followed by 30 cycles of amplification (94°C for 30 seconds, 60°C for 30 seconds, 72°C for 3 minutes) and a final extension at 72°C for 5 minutes. PCR products were gel purified using the QIAquick gel extraction kit (Qiagen, 28704) and sequenced at the Biopolymers Laboratory at the Massachusetts Institute of Technology.

## Supporting Information

Data S1 **List of coordinates and genes in the duplicated strains which underwent paired-end sequencing (M41, M141, CDC606), their intersection, and hypergeometric enrichment tests for functional significance.**
(XLS)

Data S2 **Failed chi-square test results (alpha = 0.05) of occurrence of SNPs inside versus outside the duplicated region for strains M41, M141, CDC606, and T67 (both sets of boundaries).**
(XLS)

Data S3 **Comparison of SNPs between duplicated strain and the non-duplicated cousin.** Comparisons were made between M41 & M73, M141 & M73, CDC606 & H37Rv, and CDC606 & M73.
(XLS)

Data S4 **List of gene IDs removed from the SNP tree involving classic drug resistance genes and members of repetitive genes families.**
(TXT)

Figure S1 **Region of deletion summary showing RD105, RD207, RD181, RD150, RD142 for strains M141, M41, T67, CDC606, H37Rv, and M73.**
(TIF)

Figure S2 **Coverage plot showing RD105: 79,567 to 83,034 spanning genes Rv0071 to Rv0074.**
(TIF)

Figure S3 **Coverage plot showing RD207: 3,120,521 to 3,127,920 spanning genes Rv2814c to Rv2820c.**
(TIF)

Figure S4 **Coverage plot showing RD181: 2,535,429 to 2,536,140 spanning genes Rv2262c to Rv2263.**
(TIF)

Figure S5 **Coverage plot showing RD150: 1,896,862 to 1,899,349 spanning genes Rv1671 to Rv1674c.**
(TIF)

Figure S6 **Coverage plot showing RD142: 1,332,182 to 1,335,033 spanning genes Rv1189 to Rv1192.**
(TIF)
