# Supplementary figures and images for: Independent Large Scale Duplications in Multiple M. tuberculosis Lineages Overlapping the Same Genomic Region
Source: PLoS One. 2012 Feb 7;7(2):e26038. doi: 10.1371/journal.pone.0026038 (PMC3274525; doi:10.1371/journal.pone.0026038)

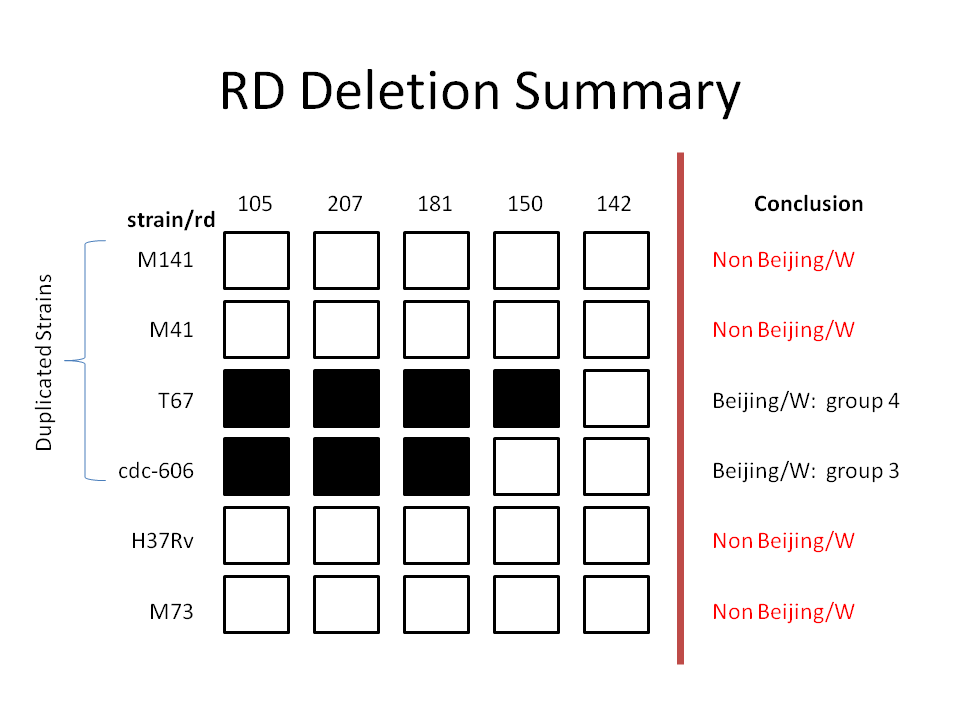

Supplement: Figure S1 — Region of deletion summary showing RD105, RD207, RD181, RD150, RD142 for strains M141, M41, T67, CDC606, H37Rv, and M73. (TIF) [file pone.0026038.s005.tif]

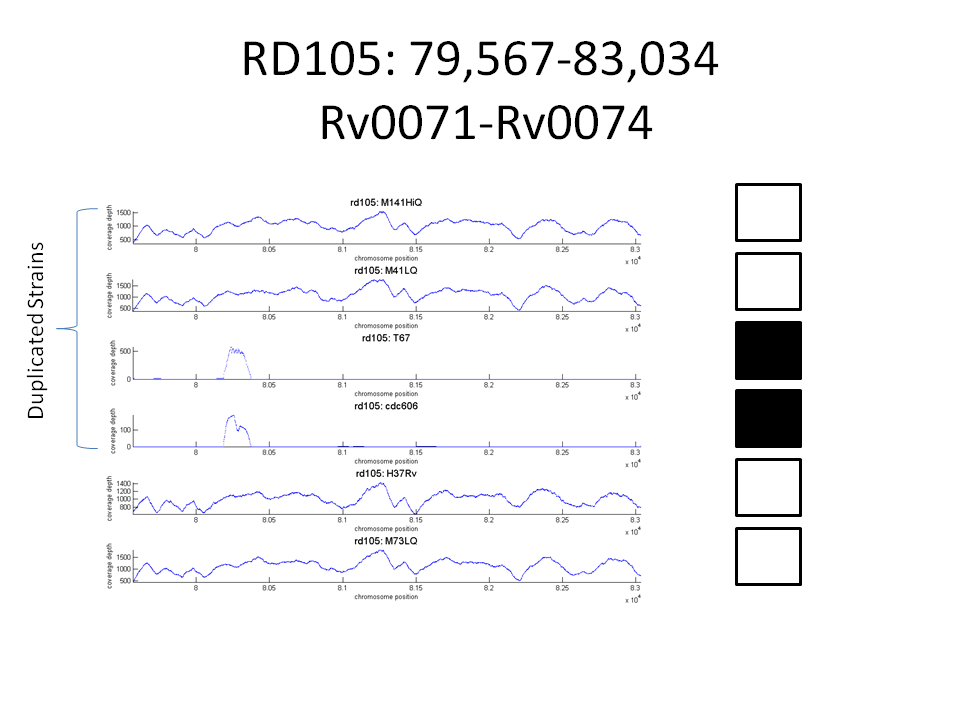

Supplement: Figure S2 — Coverage plot showing RD105: 79,567 to 83,034 spanning genes Rv0071 to Rv0074. (TIF) [file pone.0026038.s006.tif]

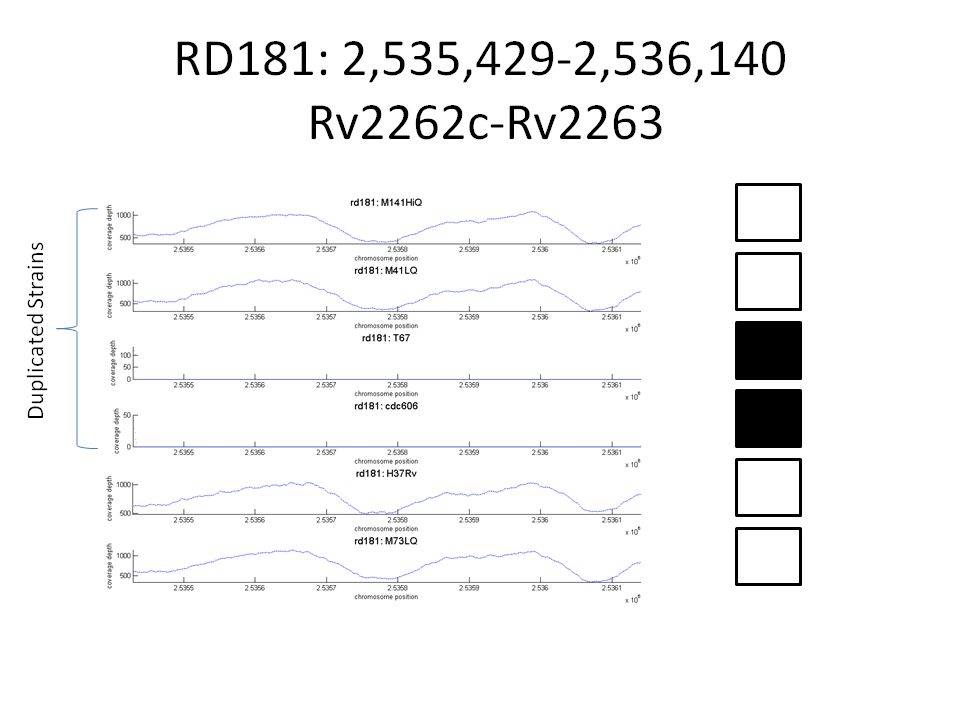

Supplement: Figure S3 — Coverage plot showing RD207: 3,120,521 to 3,127,920 spanning genes Rv2814c to Rv2820c. (TIF) [file pone.0026038.s007.tif]

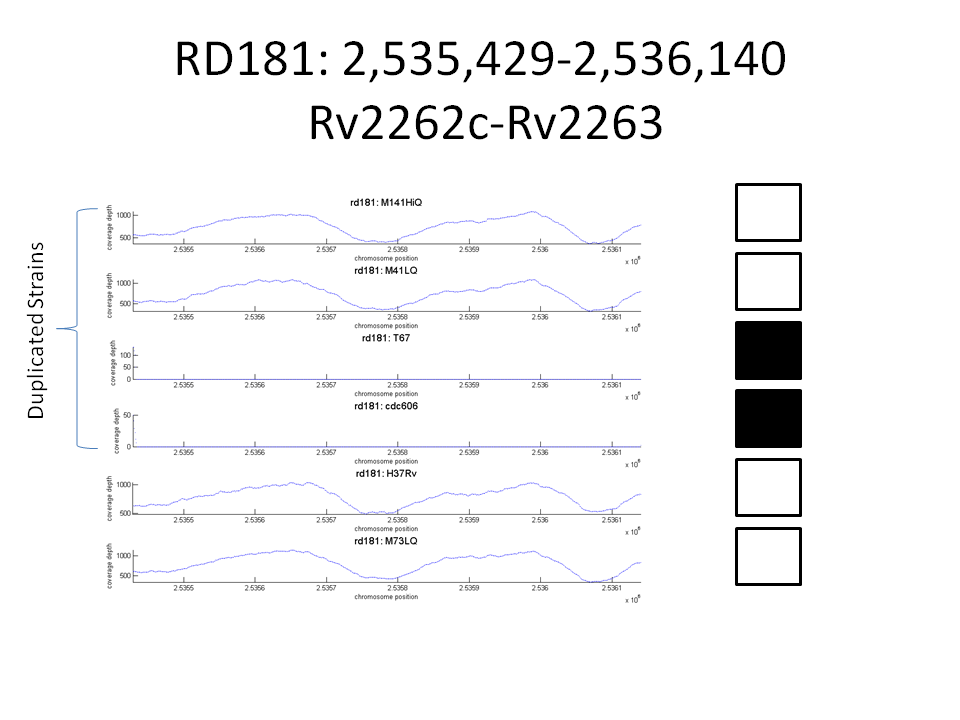

Supplement: Figure S4 — Coverage plot showing RD181: 2,535,429 to 2,536,140 spanning genes Rv2262c to Rv2263. (TIF) [file pone.0026038.s008.tif]

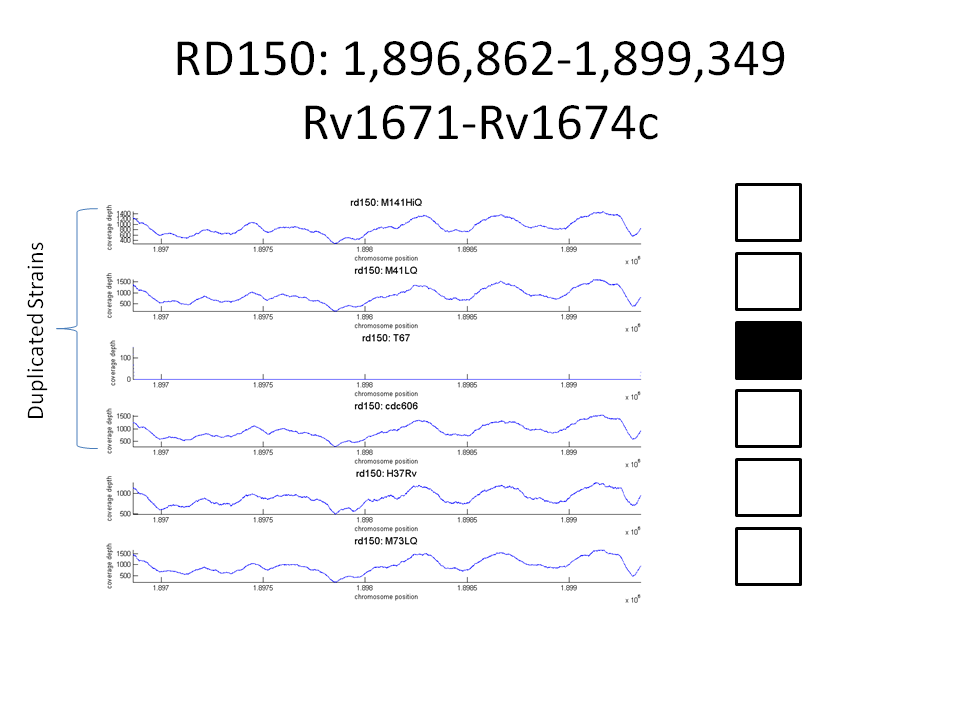

Supplement: Figure S5 — Coverage plot showing RD150: 1,896,862 to 1,899,349 spanning genes Rv1671 to Rv1674c. (TIF) [file pone.0026038.s009.tif]

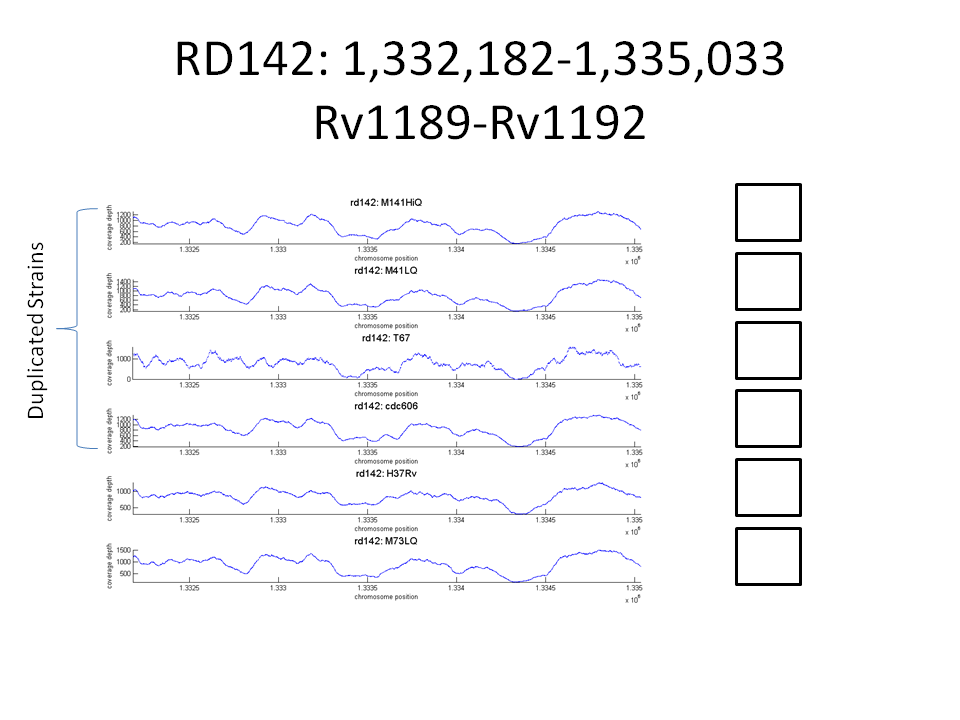

Supplement: Figure S6 — Coverage plot showing RD142: 1,332,182 to 1,335,033 spanning genes Rv1189 to Rv1192. (TIF) [file pone.0026038.s010.tif]
